# Enhancing reflective practice through online learning: impact on clinical practice

**DOI:** 10.2349/biij.4.1.e8

**Published:** 2008-01-01

**Authors:** J Sim, A Radloff

**Affiliations:** Medical Radiations, School of Medical Sciences, RMIT University, Victoria, Australia

**Keywords:** Education, reflective practice, clinical practice, online learning

## Abstract

**Purpose:**

Traditionally, radiographers and radiation therapists function in a workplace environment that is protocol-driven with limited functional autonomy. The workplace promotes a culture of conformity and discourages practitioners from reflective and critical thinking, essential attributes for continuing learning and advancing workplace practices. As part of the first author’s doctoral study, a continuing professional development (CPD) educational framework was used to design and implement an online module for radiation therapists’ CPD activities. The study aimed to determine if it is possible to enhance healthcare practitioners’ reflective practice via online learning and to establish the impact of reflective learning on clinical practice.

**Materials and methods:**

The objectives of the online module were to increase radiation therapists’ knowledge in planning for radiation therapy for the breast by assisting them engage in reflective practice. The cyclical process of action research was used to pilot the module twice with two groups of volunteer radiation therapists (twenty-six participants) from Australia, New Zealand and Canada.

**Results:**

The online module was evaluated using Kirkpatrick’s four-level evaluation model. Evidence indicated that participants were empowered as a result of participation in the module. They began reflecting in the workplace while assuming a more proactive role and increased clinical responsibilities, engaged colleagues in collaborative reflections and adopted evidence-based approaches in advancing clinical practices.

**Conclusion:**

The study shows that it is possible to assist practitioners engage in reflective practice using an online CPD educational framework. Participants were able to apply the reflective learning they had developed in their workplace. As a result of their learning, they felt empowered to continue to effect changes in their workplace beyond the cessation of the online module.

## INTRODUCTION

Traditionally, Medical Radiation Science (MRS) practitioners (radiographers and radiation therapists) function in a workplace environment that is protocol-driven and has limited functional autonomy [1-4]. The workplace promotes a culture of conformity and discourages practitioners from reflective and critical thinking, essential attributes for continuing learning and for advancing workplace practices. Although such a workplace culture promotes development of competent clinical practitioners, it will not lead to reflective-thinking practitioners.

Reflective thinking can assist MRSpractitioners in their current roles. In a workplace that is protocol-driven, reflective thinking can assist practitioners to break away from the protocol-driven workplace culture [5]. Reflective thinking empowers practitioners by highlighting best practice that enhances clinical performance, thereby increasing professional self-esteem [6]. Knowledge that is empowering and satisfying is locally generated and attained via reflective dialogues conducted with peers [6]. Reflective thinking empowers MRS practitioners to move beyond a subservient mindset and conformity while motivating them to continue learning [7]. Reflective practitioners assume responsibility for their own learning, are open to new ideas and constantly seek to advance workplace practices [8-10]. They collaborate with their peers and adopt a holistic approach towards problem solving [8]. They engage in critical reflection of their practice and examine their values, leading to transformative learning that not only transforms perspectives of themselves as healthcare practitioners but also results in new insights into their practice [7-8, 11-12].

The current focus of continuing professional development (CPD) programs has been on updating practitioners’ clinical knowledge [13-15] with little emphasis on assisting practitioners to develop the attributes that are necessary for reflective practice and advancing clinical practice [14,16]. As part of the first author’s doctoral study, an online module was designed using a CPD educational framework and implemented as part of radiation therapists’ CPD activities. The study aimed to determine if it is possible to enhance healthcare practitioners’ reflective practice via online learning and to establish the impact of reflective learning on clinical practice.

## LITERATURE REVIEW

### Educational framework underpinning the online module

Constructivism, learner-centred teaching and situated learning are three major learning and teaching approaches that form the educational framework for CPD in this study, with reflective thinking chosen as one of the main learning strategies for CPD learning.

Constructivist learning requires learners to integrate past experiences with current experiences in order to make sense of their own learning [17]. Instead of memorising and regurgitating facts, learners question, analyse, negotiate and construct their own knowledge. Social constructivism involves the construction of knowledge through collaborative learning with learners pursuing shared learning goals [18-19]. Collaborative learning promotes reflection, since learners are required to discuss, explain and defend ideas thereby assisting them to reflect and to improve on their own understanding. Learners are exposed to multiple perspectives, making the resultant learning broader than what would have resulted from individual learning [20-21].

In learner-centred teaching, the emphasis is on the process of learning and on developing learners’ competence, as opposed to just knowledge acquisition based on teacher-centred teaching [22]. This shift of responsibility and power from teacher to learners is consistent with the current focus on lifelong learning, which places greater emphasis on learners assuming more responsibility for their own learning [23].

While much of the formal learning that occurs in institutions is decontextualised [24], situated learning focuses on the social and cultural aspects of learning, making learning an authentic and meaningful experience [25-27]. Situated learning involves engaging “learners in tasks that reflect practices encountered in professional workplace settings” [28]. Here, knowledge and skills are best learned by reflecting on how they are applied in everyday situations [27]. Thus, situated learning is particularly suitable for CPD programmes.

These approaches to learning shaped a learning environment that is constructive, socio-culturally mediated, learner-centred and authentic. The instructional frameworks of Salmon’s 5-stage model and constructive alignment helped to achieve the aims of the CPD program.

A feature of good education design is to create and facilitate a supportive learning environment that enables learners to engage in meaningful learning in a structured manner [29]. Salmon’s 5-stage model of teaching and learning online provides an appropriate instructional framework in guiding participants through their online learning [30-32]. Stage 1 (access and motivation) focuses on getting learners to familiarise themselves with the online learning environment, in preparation for their active participation in subsequent activities [31-33]. Stage 2 (online socialisation) is concerned with establishing the trust and repertoire between learners in order to lay the foundation for future collaborative work [31]. The next three stages are the most “productive and constructive for learning and teaching purposes” [33]. Stage 3 involves information exchange among learners, and between learners and the moderator, based on their pre-existing knowledge and the online resources made available. The role of the moderator is to maintain an intellectual role, guiding and extending the discussions, facilitating learning by providing timely feedback, suggesting resources and encouraging learners to reflect on their work [34-36]. The moderator also steers the discussions by providing prompts and initiating questions, teasing out multiple perspectives, commenting on the adequacy and quality of discussions, and if need be, challenges their contributions in a supportive, encouraging manner and within the stipulated time frame [21,31,33,35]. In Stage 4, learners start to engage in more active learning, consider multiple perspectives through negotiation and deliberation with their online peers, and often assume the role of knowledge constructors rather than mere assimilators of knowledge [31,33]. By the time learners reach the final stage (development), they are usually ready to engage in constructivist learning, becoming more critical and self-reflective. By the final stage, learners have constructed their own understanding gained from the extended debate and discussions through the previous stages and are able to function as independent learners [31,33].

**Figure 1:**
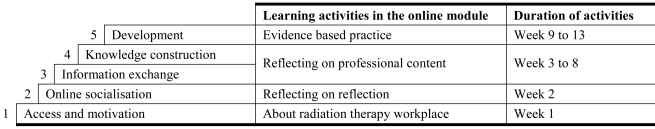
Summary of learning activities in the online module using Salmon's 5-stage teaching and learning model. [Source: Adapted from 34 p.11]

Salmon’s model provides a structured learning environment, which is sufficiently flexible to allow the education designer to design the course to meet specific educational goals. Thus, in the MRS online module, while adhering to the Stage 1 and Stage 2 of Salmon’s model, the first author contextualised the Stages 3, 4 and 5 as shown in Figure 1.

### Action Research

Action research was the research methodology used in this study. According to Frost, “Action research is a process of systematic reflection, enquiry and action carried out by individuals about their own professional practice” [37]. In action research, practitioners are no longer “objects” to be studied, but assume the role of contributors [38]. This inclusive approach reduces researchers’ personal biases and is a useful way of informing research [38]. Dick further defines action research as a “flexible spiral process which allows action (change, improvement) and research (understanding, knowledge) to be achieved at the same time” [39]. Action research narrows the gap between theory and practice and, by alternating between action and critical reflection, leads to improved practice through progressive accumulation of practical knowledge [38-39]. In this study, the cyclical process of action research provided an ideal mechanism to design, implement, evaluate, reflect on and modify the educational framework used to guide the design and development of an online CPD module for MRS practitioners. Data collected during the action research cycle were analysed and reflected on [40].

## METHODS

This study consisted of two major phases. The First Research Phase included the literature review and data collection. While the aim of the literature review was to assist in the design of the educational framework [41], the purpose of the data collection was to seek input from the clinical workplace in terms of practitioners’ learning needs. Data collection strategies included a national CPD survey for MRS practitioners and semi-structured interviews with Heads of Clinical Departments.

Based on reflection on the First Research Phase, the Second Research Phase aimed to develop an online module based on the CPD educational framework. This paper focuses on the Second Research Phase. In line with the participatory nature of action research [38], other MRS stakeholders collaborated with the first author in piloting the online module. These stakeholders included two senior radiation therapists and one MRS academic staff who assumed the role of facilitators in supporting and facilitating learning amongst participants of the online module. The online CPD module was first piloted with a group of 12 radiation therapists from Victoria and Tasmania (1^st^ pilot module), with the researcher reflecting on and using the feedback to evaluate and refine the module, which was piloted again with a second group of 14 radiation therapists from Australia, New Zealand and Canada (2^nd^ pilot module).

For the 1^st^ pilot module, recruitment of participants was via publicity pamphlets sent to major radiation therapy centres and satellite branches in Victoria and Tasmania. Due to the uncertain level of responses, selection was based on a first come first served basis and limited to one participant per centre or two participants from a larger clinical centre. For the 2^nd^ pilot module, participation was opened to radiation therapists from Australia. Volunteers were called for in the Australian Institute of Radiography National Conference in Cairns, Australia. Clinical educators from New Zealand and Canada who were attending the Conference approached the first author expressing interest for their staff to participate. Also included were participants from Canada and New Zealand who would give the online module an international dimension and be in line with the internationalisation of higher education. In an effort to accommodate more Australian participants, the total number of participants was increased from 12 to 14, resulting in eight Australian and six international participants.

### Online module

The two main learning objectives of the online module were to increase practitioners’ knowledge of radiation therapy planning and to enhance participants’ ability to reflect in the workplace. One of the prerequisites for an effective online program is the adoption of constructive alignment [42-43] where learning objectives are aligned with the learning activities and assessment tasks [44]. Given that one of the main learning objectives is reflection, it follows that the learning activities would require participants to engage in a variety of reflection activities, including reflecting on radiation therapy literature and their planning practices, and engaging in reflective dialogues with their online peers. Learning outcomes in terms of radiation therapy knowledge and reflection were assessed through participants’ reflective dialogues and activities, and evidence-based practice (EBP) assignments, and from the impact of participants’ reflection on their workplace practices.

Salmon’s five-stage framework was modified and applied to the online module. (Table 1) provides a summary of the learning activities. The first two weeks of the module focused on participants knowing their online peers and familarising themselves with the learning environment. Week 3 to 8 involved information exchange and knowledge construction. Information exchange involved participants sharing the ‘what, why and how’ of their protocols with their online peers, thereby providing the foundation for reflection and consolidation of knowledge. With knowledge construction, participants were required to read, reflect and respond to their peers on a series of nominated articles that were selected by the facilitators. In the facilitators’ personal reflective pieces and group discussions that followed, the facilitators shared why they chose the articles and how the articles impacted on their clinical practice. This first set of reflective readings was followed by a second series of articles, which was selected by the participants from the electronic database. Each participant was required to share his or her personal reflection on the chosen article, including the article's impact on clinical practice and/or how the article had further prompted more questions. Each of the week’s activities was rounded off with online personal and group reflection. Guidelines and examples were provided by the facilitators to assist participants in these reflective activities. There were also guidelines to prompt participants on the reflective process and the possible reflection outcomes that follow. Although participants were required to read a series of articles during Week 3 to 8, the learning process went beyond that of didactic delivery. The focus was on the personal and collaborative reflections that ensued rather than the selection of the ‘best’ literature. The final four weeks of the module enabled participants to put their reflection and information literacy skills into practice by applying EBP at their workplace. The EBP activity was planned with the aim of enabling participants to see how their newly acquired skills of information literacy and reflection can be successfully applied in the workplace. However, due to time and workplace constraints, participants’ EBP outcomes were demonstrated in the form of an EBP assignment.

**Table 1:** Online module learning activities

**Week**	**Topics**	**Learning Activities**
1	Getting to know one another	• Self-introduction • Sharing workplace and prior online learning experience • Reflecting on Week 1 learning
2	Professional networking	• Sharing motivation about learning • Reading, reflecting and responding to reflection literature • Sharing about reflection in the MRS workplace • Reflecting on Week 2 learning
3	Role of radiation therapy in the management of breast cancer	• Information exchange: • Sharing workplace protocols: why, what & how • Knowledge construction: • Literature search • Sharing of recommended literature • Reflecting on each topic learning
4		
5	Current planning practices for breast cancer	
6		
7	Tattoos or skin marks?	
8		
9	Applying evidence-based practice in Radiation Therapy	• Reading, reflecting and responding to EBP literature • Selecting EBP topic • EBP assignment • Sharing EBP assignment
10		
11		
12		
13	Final reflection and celebration!	• Reflecting on EBP learning • Reflecting on 13 weeks of learning

### Evaluation

Kirkpatrick’s four-level evaluation model was used in evaluating the online module. Kirkpatrick’s model focuses on the quality, efficiency and effectiveness of educational programs [45-47]. Due to its simplicity and practicality, Kirkpatrick’s model is also a useful evaluation model for online learning [48]. The model allows the evaluation of participants’ reaction to the program (Level 1), participants’ learning (Level 2), behavioural change as a result of participation in the module (Level 3) and evaluation of the impact of participation in the workplace (Level 4) [49-50]. Due to the constraints of this paper, it is not possible to present all the evaluation criteria for all four levels of evaluation. Given that, this paper is about reflective practice and its impact on clinical practice with focus on ***reflection outcomes*** in terms of participants' learning (Level 2), behavioural changes (Level 3) and impact in the workplace (Level 4).

Data were collected from multiple sources using both quantitative and qualitative approaches, a combination common in action research [40,51-52]. Quantitative approaches included pre-, mid- and post-module surveys while qualitative approaches included participants’ postings at online discussion forums and learning portfolio, and minutes of the researcher’s (first author) meeting with facilitators, as well as the first author’s reflective journal. Quantitative data provides a summarised and condensed form of data while qualitative data enhances the data by demonstrating the links between complex and large amounts of data [53]. Thus, while quantitative data is useful in presenting an overall picture and snapshot of a particular phenomenon, qualitative data is able to provide further descriptive details as to the reason(s) for the phenomenon depicted. In this instance, the qualitative data collected from multiple sources contributed towards providing a clearer picture to the links between Level 2 (participants' learning), Level 3 (behavioural changes) and Level 4 (resultant impact in the workplace) data. In addition, the use of multiple data collection strategies allows cross-data validity checks, thereby increasing the rigour, validity and credibility of the findings [52,54-57]. (Table 2) summarises the data collection strategies used for each level of evaluation, with participants, participants’ workplace supervisors, facilitators and the first author contributing to the data.

**Table 2:** Kirkpatrick’s four level evaluation model and corresponding data collection strategies

**Evaluation level**		**Data collection strategies**
1	Reaction data	Mid module survey Post module survey Messages posted at discussion forum
2	Learning data	Pre-module survey and Post module survey Content analysis of reflection postings via Boud et al framework Content analysis of other learning outcomes via learning objectives of online module Facilitators’ reflective journals Participants’ learning portfolio
3	Behavioural data	Workplace survey (to be completed by Supervisor) EBP assignment assessment Messages posted at discussion forum 3-month post module survey
4	Impact data	Workplace survey 3-month post module survey Learning portfolio of participants Continuing communication with participants

Quantitative data was analysed using SPSS for MS Windows Version 13.0 while qualitative data was coded and analysed with Nvivo7. Coding qualitative data provides a framework for subsequent data analysis, enabling data triangulation and interpretation and conclusions to be drawn [57]. Qualitative data were coded using Henri’s thematic unit of analysis [58-59].

Meaningful evaluation is only possible when there is good understanding and successful incorporation of appropriate pedagogy into evaluation strategies [60]. Thus, an appropriate conceptual model of the reflective process is needed to inform and guide the researcher as to the criteria for analysing and evaluating the data obtained from online discussions [61]. The evaluation criteria for reflection outcomes were based on a paper by Boud et al on reflective model and a paper by Gunawardena et al. on social construction model on computer-mediated communication. Consequently, seven levels of reflective process for coding were identified [62-63].

## RESULTS

The online module had two main objectives; to enhance practitioners’ ability to reflect and to increase their radiation therapy knowledge. The third main objective of empowering participants was not made known to the participants to avoid the possibility of tainting participants’ reporting of learning outcomes.

The completion rate for the 1^st^ and 2^nd^ pilot was 58% and 71%, respectively. For both pilot modules, there was a good spread of participants both in terms of age group and years of experience (see (Table 3)). The 2^nd^ pilot participants were much more proactive than the 1^st^ pilot participants in exchanging and exploring issues raised in the discussion forum. This was evidenced from the higher number of messages posted in the 2^nd^ pilot. The inclusion of the international participants in the 2^nd^ pilot module has also contributed to the increased exchanges as participants were keen to find out if radiation therapy practices differ between countries.

**Table 3:** Demographics of participants from 1st and 2nd pilot

	Age group			
	20-29	30-39	40-49	50+
Percentage	23.1	26.9	34.6	15.4
	Number of years of radiation therapy experience			
	Less than 5	5-10	10-19	20+
Percentage	26.9	23.1	19.2	30.8

* n = 26.

Data collected show that learning outcomes included participants’ increased understanding of radiation therapy, motivation for learning and sharing their learning with colleagues, confidence as self-directed learners, information literacy skills and understanding of EBP. For the purpose of this paper, the authors focused on reflection outcomes (Level 2 and Level 3 data) and the impact of learning on clinical practice (Level 4 data).

## PARTICIPANTS’ REFLECTION OUTCOMES

### Level 2 Evaluation: Learning data

In this study, Boud et al’s (1985) reflective model was used as the conceptual framework for coding and evaluation of reflection and learning outcomes. Boud et al proposed a generic framework of reflection that describes seven levels of reflection processes that learners might experience [62]. The foundation level includes returning to experience, which involves describing the activities, an essential step of recounting past experiences so that subsequent reflections are based on actual recollection of events. Attending to feelings (1^st^ level) recognises the importance of feelings in facilitating or obstructing the learner’s learning experience since “utilizing our positive feelings is particularly important as they can provide us with the impetus to persist in what might be very challenging situations” [62]. Allowing learners to articulate their feelings assists them in understanding their emotions in the learning context, an important characteristic of the self-directed learners [64]. The 2^nd^ to 5^th^ levels consist of association, integration, validation and appropriation. Association (2^nd^ level) refers to relating new knowledge to pre-existing understanding, integration (3^rd^ level) involves synthesising old and new data, while validation (4^th^ level) is “testing for internal consistency” including the testing of new concepts [62]. Finally, appropriation (5^th^ level) involves internalising knowledge into one’s cognition. These levels do not necessarily occur in sequence, neither do learners need to experience each level of reflective process described. In fact, validation and appropriation, which form the higher level of the reflective process, could also be viewed as a form of reflective outcomes. Reflective outcomes (6^th^ level) ranged from changes in behaviour (action outcomes), changes in the learner’s affective state (affective outcomes) and/or perspectives (perspectives outcomes) [62] (see Tables 4 and 5).

**Table 4:** Level 2 learning data: Coding results of reflection outcomes for 1st pilot participants

**Level of reflective process**	**Code**	**1st pilot participants**							
		**1**	**2**	**4**	**7**	**8**	**11**	**12**	**Total**
Returning to experience: Sharing and exchanging information	0	2	5	3	2	5	11	7	35
Attending to feelings	1								
• Positive feelings	1A	3	-	-	-	-	-	1	4
• Negative feelings	1B	1	-	-	-	-	-	-	1
Association	2	2	3	5	2	3	4	2	21
Integration	3	1	1	-	2	1	2	1	8
Validation	4	-	-	-	-	-	1	-	1
Appropriation	5		-	-	-	-	-	-	-
Outcomes of reflection	6								
• Action	6A	3	3	3	2	3	2	4	20
• Affective (emotions)	6B	-	-	-	2	-	-	1	3
• Perspectives	6C	3	1	-	1	-	-	1	6
**Total**		**15**	**13**	**11**	**11**	**12**	**20**	**17**	**99**

* The left hand column lists the reflection evaluation criteria while the numerals represent the number of reflection outcomes evidenced from each participant’s contributions in the discussion forums.

**Table 5:** Level 2 learning data: Coding results of reflection outcomes for 2nd pilot participants

**Level of reflective process**	**Code**	**2nd Pilot Participants**										
		**1**	**2**	**4**	**5**	**8**	**9**	**10**	**11**	**12**	**14**	**Total**
Returning to experience: Sharing and exchanging information	0	9	12	11	15	11	7	9	18	5	2	99
Attending to feelings	1											
• Positive feelings	1A	2	1	-	2	1	2	2	2	1	2	15
• Negative feelings	1B	2	1	1	-	-	1	1	-	1	1	8
Association	2	4	1	2	3	1	1	2	5	-	1	20
Integration	3	-	1	1	1	-	2	1	2	-	-	8
Validation	4	-	-	-	-	-	-	-	-	-	-	-
Appropriation	5	-	-	-	-	-	-	-	1	-	1	2
Outcomes of reflection	6											
• Action	6A	2	2	4	2	1	1	1	6	4	2	25
• Affective (emotions)	6B	-	-	-	-	-	-	-	1	-	1	2
• Perspectives	6C	-	-	1	-	2	-	-	3	2	1	9
**Total**		**19**	**18**	**20**	**23**	**16**	**14**	**16**	**38**	**13**	**11**	**188**

* The left hand column lists the reflection evaluation criteria while the numerals represent the number of reflection outcomes evidenced from each participant’s contributions in the discussion forums.

All participants in both pilot modules reported the initial level of describing, sharing and exchanging information and association. Most demonstrated integration, with only two participants from both pilot modules showing evidence of validation and appropriation. One possible reason why few participants showed the higher levels reflective processes of validation and appropriation could be that the coded data only captured the end process of reflection rather than the continuum of participants’ reflection.

As a result of the reflective dialogues and activities, all participants had at least one coding that demonstrated an action outcome of reflection activities in the module. In terms of reflective outcomes, most of the outcomes came under the action category. This finding refers to explicit expressions by participants about their commitment to action. The action assumed the form of participants using their newly acquired knowledge, applying their reflective and/or information literacy skills, with the ultimate aim of initiating new projects, or assessing and suggesting changes to their workplace practices, as illustrated by the following comments:

I am now confident in knowing where to search for information and I have lots of little projects that I can do in mind. [Participant 12: 1^st^ Pilot] (Note: ‘Participant 12: 1^st^ Pilot’ refers to Participant number 12 from 1^st^ pilot module)

I hope I can look at practices in our department and use some of the knowledge gained to assess and maybe even change! [Participant 12: 2^nd^ Pilot]

### Level 3 Evaluation: Behavioural data

Level 3 refers to participants' behavioural change as a result of participation in the module. These changes ranged from changes in radiation therapy practice to changes in attitude and behaviour in the workplace.

Data from the Workplace Survey, the 3-month post-module survey and the EBP assignments were used to establish behavioural changes. The response rate for the Workplace Survey was 71% and 40% for the 1^st^ and 2^nd^ pilot modules, respectively. With the exception of two participants who were reported to have shown no change, responses from the Workplace Survey showed evidence that participants were empowered as a result of participating in the modules. Their Supervisors reported them to be enthusiastic with increased confidence and they displayed a positive attitude at work and towards learning. Changes in the form of radiation therapy planning included an appreciation of the complexities of radiation therapy planning with three participants continuing to implement changes and improvements to their planning as discussed in the online forum. Other changes included engaging in literature search, assisting colleagues with online searches and actively seeking for new challenges at work.

The 3-month post-module survey showed that for both the 1^st^ and 2^nd^ pilot modules, approximately 53% of the participants continued to reflect on the literature and engage in reflective practice in the workplace. Another 53% continued to work towards their EBP activities by either presenting their EBP assignments or investigating various techniques to advance clinical practice.

### Level 4 Evaluation: Impact on clinical practice

Level 4 evaluation refers to the participants’ impact on the workplace as a result of their learning [46]. Data collection methods included the Workplace Survey, the 3-month post-module survey and the participants’ learning portfolio. Examples of Level 4 success in the MRS workplace include practitioners' advancing workplace practices, varying workplace protocols to better treatment plans, and the intangibles such as increased confidence, enthusiasm and positive attitude.

All participants in both pilot modules were unanimous that the learning experience had a positive impact on their professional development. The common response was that the module has given them the added confidence in attempting new initiatives in their workplace as evidenced by the following comments:

Participation in the module has given me greater confidence [sic] in my skills and this in turn has led to taking on greater responsibility. I’m starting to check treatment plans for the first time and acting as a senior, which I didn’t think was ever going to happen! [Participant 11: 1^st^ Pilot, 3-month post-module survey]

I have more confidence in my ability to do things on a professional level. I have been more keen to do a paper, and to attend conferences and seminars, and also to eventually have more involvement when we get our new equipment. [Participant 11: 2^nd^ Pilot; 3-month post-module survey]

In the Workplace Survey, 35.3% of the participants who successfully completed the two pilot modules were reported by their supervisors to have made a positive impact on their workplace. This impact ranged from assuming an infectious attitude towards learning, willingness to share new ideas and solutions, willingness and ability to contribute towards departmental projects such as quality improvement studies and information technology developments. These participants were also proactive in advancing radiation therapy technique development in their workplace.

In particular, reports from the Workplace Survey were outstanding for two participants. The attitudinal and behavioural change of Participant 10 (from the 2^nd^ pilot module) in embracing challenges was noted by her Supervisor:

I have noticed a change in Participant 10’s enthusiasm, towards RT planning. She is keen to learn (almost demands to learn new methods). This contrasts the way she was. She previously used to be a bit more apprehensive when challenged. [Supervisor of Participant 10: 2^nd^ Pilot]

The change in attitude and enthusiasm has in turn brought about a positive learning culture in her workplace, as the following comment illustrates:

Participant 10 is a good role model in the workplace. Her positive attitude and willingness to learn of late has had a positive impact. Especially on the more junior staff and students. [Supervisor of Participant 10: 2^nd^ Pilot]

From ongoing communication with the participant, the first author is aware that she was promoted to the position of Deputy Head a year after completing the online module.

Participant 12 from the 1^st^ pilot module was instrumental in assisting the department in proposing changes to her Headquarter Clinical Planning Committee, as illustrated by the following comment:

[Participant 12] has been able to make evidence-based suggestions with regards to our current practices and propose changes and present ideas to staff in meetings. [Supervisor of Participant 12: 1^st^ Pilot]

Participant 12 has assumed an active role in disseminating information she learned from the module through her department’s journal clubs, as well as making herself available to assist her colleagues in their online research activities. The supervisor concluded with the following comment:

I believe [Participant 12] has benefited both professionally and personally from the module. Her willingness to communicate ideas and source solutions has improved and her confidence in what she is doing has also increased. [Supervisor of Participant 12: 1^st^ Pilot]

Participants’ learning also flowed to the workplace. The three-month post-module survey showed that more than half the participants continued to read and reflect on the literature, and to engage in some form of EBP work such as exploring their EBP topic or choosing a new clinical issue for investigation. In terms of the reflection in the MRS workplace, the ongoing discussions between participants and their colleagues have certainly raised their awareness of the importance of reflection.

## DISCUSSION

### Developing a culture of reflective practice in the workplace

Developing a culture of reflective practice in the workplace does not occur spontaneously or overnight. Rather, the culture of reflective practice begins with each practitioner reflecting at an individual level and at a collective level. In terms of the latter, the participants reflected collaboratively with their online peers as well as with their colleagues in the MRS workplace.

At an ***individual level***, it is imperative that practitioners themselves are aware of what constitutes reflection, and the importance and value of reflecting at the workplace. The learning activities provided opportunities for participants to reflect on the meaning of reflection, the risks associated with reflection, and the value of reflecting in the MRS workplace. The online module was successful in raising participants’ awareness and understanding of the importance of reflecting in the workplace. This is evident from how participants shared their greater appreciation of reflection and their willingness to apply reflection in the workplace, as illustrated by the following comments:

I must confess that I have usually taken reflecting for granted, which generally means that you only revise and analyse situations and events that have had some major impact on you. From doing this module so far I have gained a new respect for the value of reflection and hope to incorporate it more in my professional and social life. [Participant 1: 1^st^ Pilot]

I think I too have taken reflection for granted. It's not something I've consciously sat down to do at work, and I tend to be one of the people that do things "because that's the way it's done." I'm hoping for this to change - in fact while thinking about our current breast planning technique I've come up with a question about tattoos I can't answer to my satisfaction. I'll quiz a few people when we return to work on Tuesday and see if I can come up with a satisfactory response!! [Participant 11: 1^st^ Pilot]

At a ***collective level*,** the culture of reflective practice in the MRS workplace was made possible when individual learning and reflection permeated the workplace. This is evident from the postings at the discussion forum, which showed that the learning and collaborative reflection were not restricted to participants’ online community but had also extended to include their colleagues in the workplace. All participants were involving their colleagues in reflective dialogues at work by sharing their reflection and literature reading, and by informing and updating them on the online discussions. Participant 8 commented as follows:

I had a lot of interest from my department in the whole idea of this type of online discussion forum. I would start talking about one topic, which may have been mentioned in the discussion forums and it sparked up further conversations. One thing with RTs [radiation therapists] there is never any shortage of opinions and passion for our work. Just sometimes there is a shortage of RTs ;-) [Participant 8: 2^nd^ Pilot]

Participants sought input from colleagues and brought the workplace discussions back to their online peers. Thus, the reflective dialogues that started on the discussion forum flowed into the workplace and then looped back to the online community, as illustrated by the comment below:

Many RTs [radiation therapists] were really interested and they really helped me look at the practices in our department. Everyone was keen to help me understand various [sic] aspects of my EBP topic and the more questions I came up with set them to thinking and reflecting [sic] on why we do things [Participant 12: 2^nd^ Pilot]

By involving their colleagues in such discussions, participants were also engaging their workplace colleagues in the Schön’s concept of reflection-on-action [65]. Such exchanges marked the beginning of a culture of reflective practice in the MRS workplace.

Another way of facilitating and spearheading a reflective culture in the MRS workplace involved participants, who themselves were supervisors in the workplace, gaining a better appreciation and understanding of reflection, as illustrated by the comment by Participant 8:

I am beginning to feel that I need to think about how I will share the ideas and information I am gaining from this experience with my workplace. With a positive approach to reflection, a workplace can grow as a unit. Through reading the article and the repsonses [sic] and thoughts posted here in the last week I also feel I have a whole new meaning for the word reflection and it doesn't [sic] involve a mirror ;-) [Participant 8: 2^nd^ Pilot]

### Impact of reflection on adherence to protocol

The ***strict adherence to protocol*** in the MRS workplace promotes conformity of practice and does not encourage regular reflection on workplace practices. Being able to question and reflect on workplace practice is certainly a departure from the entrenched MRS culture of protocol. This explains why Heads of Department (HOD) interviewed in the First Research Phase spoke of the importance of promoting and encouraging MRS practitioners to engage in EBP as “evidence-based practice goes towards benefiting your workplace” [HOD7]. Of the eight HOD interviewed, half specifically indicated that EBP is a useful avenue for introducing practitioners to questioning workplace practices and research.

So how did engaging in reflection in the online module change participants’ adherence to workplace protocol? Data showed that the online discussions and EBP activities were successful in getting practitioners to question and reflect on their workplace practices. For instance, as a direct result of the reflective dialogues on planning practices, Participant 11 spoke of how she took the initiative during one of her planning to modify the protocol, resulting in a 10% reduction in radiation delivered to the patient, as the comment below illustrates:

Although the plan I had produced was "acceptable" - I asked the senior RT checking my plan if it would be considered going "over the top" to add a lightly weighted 18X beam on the lateral to further reduce the hotspot in the axilla…It's such a grey area though - if I hadn't asked, the plan would have been accepted, and the patient would be getting an extra 10% in the axilla. If I'd asked another RT , they may have thought the extra work required did not justify the end result. Maybe not... I'm just trying to think of alternatives! [Participant 11: 1^st^ Pilot]

The impact of the EBP assignment on the protocol-driven culture is also evidenced by participants’ adoption of a more critical approach at work, as illustrated by Participant 4’s comment below:

I definitely have gained a lot from doing this assignment and the module. In relation [sic] to my assignment [sic], I planned to treat? a young lady today and decided to omit the wedge on the medial field, using instead a larger wedge on the lateral and adjusting the weightings. Also, the module has helped me look a lot more laterally at things and be more inquisitive. [Participant 4: 2^nd^ Pilot]

Facilitator 2 also noted in his reflective journal how the online reflective course has impacted on workplace practices, as illustrated by the following comment:

It was extremely rewarding, however, to note that a simple point like not including medial wedges on tangents has emanated from this module and already impacted on department’s practice around the world. Very cool to think, that patients are directly benefiting from this module, with feedback from participants. [Facilitator 2]

Participant 1 gave an example of how her enhanced critical thinking had allowed her to be more proactive in advancing patient care, as illustrated by the following comment:

I think working on the EBP (which I can't seem to get finished) has got me thinking more critically about other issues with breast and other treatments at work. Last week I was working on a new machine with dynamic wedging and MLC, where you would expect less scatter from the machine ... and I noticed a remarkable number of breast patients with a brisk skin reaction - something I haven't been seeing elsewhere. Despite having seen these patients daily, the usual staff on that machine wasn’t concerned, and didn't notice a trend. I did mention it to our physicists however, to see if there could be an explanation. We're planning on watching for notable skin reactions for patients on the new machines versus old, and we may do some TLD measurements to check the skin doses on patients with bad reactions. This is something I probably wouldn't have pursued before doing this module and project! [Participant 1: 2^nd^ Pilot]

### Perspective transformation: Impact on workplace practice

***Perspective transformation*** is only possible if practitioners are given the opportunity to construct and de-construct the social context in which they work. Participant 12 was one of the participants who experienced perspective transformation. Realising that EBP is not just the responsibility of oncologists, but also of the radiation therapists, Participant 12 was able to demonstrate in her EBP assignment her understanding of the challenges facing EBP implementation in her workplace. Facilitator 2 was particularly impressed with her perspective transformation, as illustrated by the following comment:

I know of Participant 12’s department. It is extremely protocol-driven, with a culture of “nati [sic]-change’. She has recognised this, is not perturbed by it, has thought through the [EBP] process required and has a great chance of implementing her change and procedure. [Facilitator 2]

Facilitator 2 also reflected on the impact this module had on another Participant:

To give an example of the impact [this module] has had on one of the participants – she now is willing to offer an opinion at the unit audit, sharing the information and knowledge that was garnered through the online module. Further example is a patient on treatment recently who was prescribed a fractionation schedule different to the norm. Participant 11 conducted a literature search, researched the basis for the fractionation, and then presented to the whole department a synopsis of the article, in an attempt to open a dialogue with the prescribing radiation oncologist. The fact that she would never have done this prior to the online module is a clear indication of the impact that it has had on her in the workplace. [Facilitator 2]

### Empowerment of participants

Another important outcome of this online module is the empowerment of participants. Providing opportunities for participants to reflect on their workplace contribution and to claim ownership of their learning allows them to be empowered in the process, as illustrated by the following comment:

It's been an incredible time - I've learnt that my opinion is valued and appreciated and that I can analyse and reflect on what I read instead of being told what to think. [Participant 11: 1^st^ Pilot]

As a result of undertaking the EBP assignment, participants realised that they were able to contribute to workplace practices, thereby making a difference in their workplace, as the comment by Participant 4 illustrates:

I have a much clearer idea of EBP now, and hope to get more opportunity to use it in the department. I find I can think of lots of projects that I want to do! I think it is possible to make a difference - most of my colleagues are reasonably open-minded. I shall be giving them a presentation on this course in a few weeks, and hopefully I shall have some results of my EBP from this project by then. [Participant 4: 1^st^ Pilot]

Empowered and with increased confidence in their ability to contribute to the workplace, participants were able to transcend their negative mindset of “I am only a radiographer” and began to be more proactive in seeing how they could contribute to advancing workplace practices. Facilitator 3 noted the effectiveness of the online module in bringing about empowerment of participants, as the following comment illustrates:

I don’t think it was until this second module that I really started to notice that participants were getting involved in the workplace and involving other staff in finding evidence to justify themselves. It was great to hear that people were beginning to realise the importance of EBP. It only felt like a small beginning, however these are often people who have not studied, let alone really been involved in facilitating change within their departments, you could see the realisation starting to occur to them that they had the knowledge and skills to bring about these important changes. The most important thing that I think I saw some people get out of the program probably was a certain level of professional self-empowerment. It was really gratifying to be a part of. [Facilitator 3]

While the MRS literature shows practitioners to be unwilling to accept increased clinical responsibility in the workplace, there is evidence that the online learning experience described in this paper has transformed participants’ attitude towards EBP while increasing clinical responsibility, as the following comment illustrates:

I didn't think much about EBP. I briefly understand what it is but thought this has more to do with the doctors where they have to keep up with all the clinical changes…I used to think it is not my problem. After reading the article, I found this is of everyone's concern. Technology is moving rapidly, if it is the doctors' [sic] responsibilities to improve the treatment outcome clinically then I guess it would be the responsibilities of the RT to ensure this happens. Stabilisation [sic] and optimisation of dose would be the areas that need to be constantly developed. Each of us has a role to play here. [Participant 12: 1^st^ Pilot]

The EBP assignment enabled participants to put into practice their learning, validating their newfound confidence.

Transformative learning is the key to empowerment. Through critical reflection and reflective discourse, participants obtained new perspectives, which enabled them to ‘think beyond the square’. Empowered and armed with newfound confidence and changed perspective, participants began pushing their professional boundary. They began to believe in their own capabilities and started to assume a more proactive role in the workplace, adopting evidence-based approach to making suggestions. Abandoning the negative mindset and subservient attitude, participants started on literature search to keep abreast of the latest RT updates, while others started participating in ongoing department projects.

The following comments encapsulate the impact of participants’ learning in the MRS workplace:

I feel like I'm in a much better position to offer input after this module. I also think I've gained some confidence in approaching our physics staff and senior RTs with ideas (i.e. removing medial wedges and moving younger patients to our newer machines). It was great to have a reminder that there are always new and interesting articles out there. Since we do work in an EBP environment, it's important that we keep up to date ourselves and not just rely [sic] on the doctors to do so. [Participant 1: 2^nd^ Pilot]

Working on the EBP assignment has been inspiring - perhaps I'll never lead a trial in a prone breast board at our department, but there are always other opportunities. At the very least as [Facilitator 2] has reminded me, I'll be doing my very first presentation to the rest of the staff about this course - who knows, you might see me at my first conference next year as a presenter! Anything's possible :) [Participant 11: 1^st^ Pilot]

## CONCLUSION

Against a workplace culture that promotes conformity and that is protocol-driven, data from the study show that the online module succeeded in assisting participants to engage in reflective practice in the workplace. Findings from this study are in line with the educational literature on reflective learning and practice. Evidence from the study shows that it is possible to bring about empowerment, transformative learning and reflection outcomes that go beyond just mere acquisition of clinical knowledge online. Most CPD programs of the profession focus on enhancing clinical competence. While reflective practice is not new to the health profession, the success of this MRS study offers a challenge to the MRS profession to embrace reflective practice and to support CPD that focuses on developing reflection. This study represents a small but significant step towards enhancing reflective practice via online learning in Medical Radiation Science.
